# The complete mitochondrial genome of the two-spotted cricket *Gryllus bimaculatus* (Orthoptera: Gryllidae) from South Korea

**DOI:** 10.1080/23802359.2021.1901617

**Published:** 2021-03-23

**Authors:** Bia Park, Eun Hwa Choi, Gyeongmin Kim, Cho Rong Shin, Jihye Hwang, Su Youn Baek, Ui Wook Hwang

**Affiliations:** a Department of Biology Education, Teachers College & Institute for Phylogenomics and Evolution, Kyungpook National University, Daegu, South Korea; b School of Life Sciences, Graduate School, Kyungpook National University, Daegu, South Korea; c Institute for Korean Herb-Bio Convergence Promotion, Kyungpook National University, Daegu, South Korea; d Biomedical Convergence Science and Technology, Kyungpook National University, Daegu, South Korea

**Keywords:** Gryllidae, *Gryllus bimaculatus*, mitochondrial genome, molecular phylogeny

## Abstract

The complete mitochondrial genome of a two-spotted cricket *Gryllus bimaculatus* (Orthoptera: Gryllidae) from South Korea is determined and characterized in this study. The circular genome is 16,075 bp long, which consists of 13 protein-coding genes (PCGs), 2 ribosomal RNA genes, 22 transfer RNA genes, and an A + T−rich region. It has a base composition of A (40.35%), G (9.09%), C (16.80%), and T (33.76%). The gene order is identical to the ancestral gene arrangement pattern generally shown in arthropods, with the exception of an inversion of *trnN*-*trnS1*-*trnE* into *trnE*-*trnS1*-*trnN*. The maximum likelihood (ML) tree supports that *G*. *bimaculatus* is a distinct member of the monophyletic family Gryllidae.

The two-spotted cricket *Gryllus bimaculatus* De Geer, 1773, one of the most abundant cricket species, is widely distributed in tropical and subtropical regions of Asia, Africa, and southern Europe (Ragge 1972; Harrison and Bogdanowicz 1995; Mito and Noji 2008). This species belongs to the family Gryllidae within the superfamily Grylloidea (Order Orthoptera), which is known as a general food ingredient in South Korea (i.e. Kim et al. 2020). Generally, *G*. *bimaculatus* can be easily distinguished from the other species belonging to the genus *Gryllus* by the two spots on the base of its wings. Recently, the complete mitochondrial genome of *G*. *bimaculatus* was reported from China (Wang et al. 2019). Here, we fully determined the mitochondrial genome of *G*. *bimaculatus* from South Korea, which was compared with that of Chinese one previously known, with examination of the phylogenetic position within the family Gryllidae.

In this study, a reared specimen of *G*. *bimaculatus* was obtained from National Institute of Agricultural Sciences, Wanju-gun 55365, South Korea (35°49′45.45″N, 127°02′27.13″E). The specimen is kept in the Kyungpook National University (KNU), Daegu 41566, South Korea (voucher no. KNU2020001) and the extracted genomic DNA is stored in the −80 °C in the same KNU depository. The genomic DNA from South Korean specimen was extracted from the whole body using a DNeasy Blood & Tissue kit (Qiagen, Hilden, Germany). The 150 bp paired-end reads were generated by Hiseq X Ten platform (Illumina, San Diego, CA) sequencing of libraries contacting inserts of ca. 520 bp. The mitochondrial genome was extracted from the whole-genome sequencing data (6.6 Gb read sequences, unpublished data) using the program DeconSeq version 0.4.3 (http://deconseq.sourceforge.net/). The raw data of mitochondrial genome were mapped and annotated using the assembler gsMapper version 2.8 (Roche Inc., Basel, Switzerland) with GenBank DB as the reference (e.g. *G. bimaculatus* [MK204367], *Velarifictorus hemelytrus* [NC030762], and *Teleogryllus emma* [NC011823]).

The mitochondrial genome of the South Korean *G*. *bimaculatus* was completely sequenced, which is 16,075 bp in length (GenBank accession no. MT993975), and contains a standard gene component set including 13 protein-coding genes (PCGs) (*cox1-3*, *cytb*, *nad1-6*, *nad4L*, *atp6*, and *atp8*), 2 ribosomal RNA genes (*rrnL* and *rrnS*), 22 transfer RNA genes, and a non-coding A + T−rich region (CR). Of the 37 typical mitochondrial genes, 21 position on the heavy strand (H-strand) and the remaining 16 on the light strand (L-strand). The overall genome components and gene order are identical to those of the Chinese *G*. *bimaculatus* published by Wang et al. (2019). In comparison with the ancestral arthropod gene order (*trnN*-*trnS1*-*trnE* on the H-strand) (Boore 1999; Woo et al. 2007; Ryu and Hwang 2010; Baek et al. 2014; Park et al. 2016), an inverted gene order (*trnE*-*trnS1*-*trnN* on the L-strand) is observed in *G*. *bimaculatus* from South Korea and China. The inverted feature is known to be commonly found in all sequenced mitochondrial genomes of the family Gryllidae (Ma and Li 2018). The base composition is 40.35% for A, 9.09% for G, 16.80% for C, 33.7% for T, respectively, which indicates that A + T content (74.11%) is apparently higher than G + C content (25.89%). In all PCGs, the five A + T-biased codons are mainly used: ATT, ATA, AAT, TTA, and AAA. The longest PCG is 1,734 bp for *nad5*, whereas the shortest 156 bp for *atp8*. The common start codon is ATT or ATG, except for *cox1* with TCG and *nad1* with TTG. TAA is the most frequent stop codon, except for the *nad3* and *nad4* with TAG. All tRNAs except *trnS1* lacked a stable dihydrouridine (DHU) arm have the typical clover-leaf secondary structure, which is a common phenomenon, generally found in metazoan mitochondrial tRNAs (Wolstenholme 1992).

To elucidate the phylogenetic position of the two-spotted cricket *G*. *bimaculatus* within the family Gryllidae, the nucleotide sequences of 13 PCGs were aligned and analyzed from 20 orthopteran mitochondrial genomes, out of which *Locusta migratoria* (Acrididae) was employed as an outgroup. The phylogenetic analysis was conducted by a maximum likelihood (ML) method in IQ-TREE web server (Trifinopoulos et al. 2016) and the best fitting model GTR + F + I + G4 was selected by ModelFinder (Kalyaanamoorthy et al. 2017). As shown in Figure 1, the South Korean and Chinese specimens of *G*. *bimaculatus* are grouped together, placing within the monophyletic family Gryllidae. It is expected that this study could be helpful in elucidating genetic diversity between two-spotted crickets in East Asia including South Korea, China, Japan, etc., and moreover in making a conservation of such edible insect species to become potential food resources for the future.

**Figure 1.**
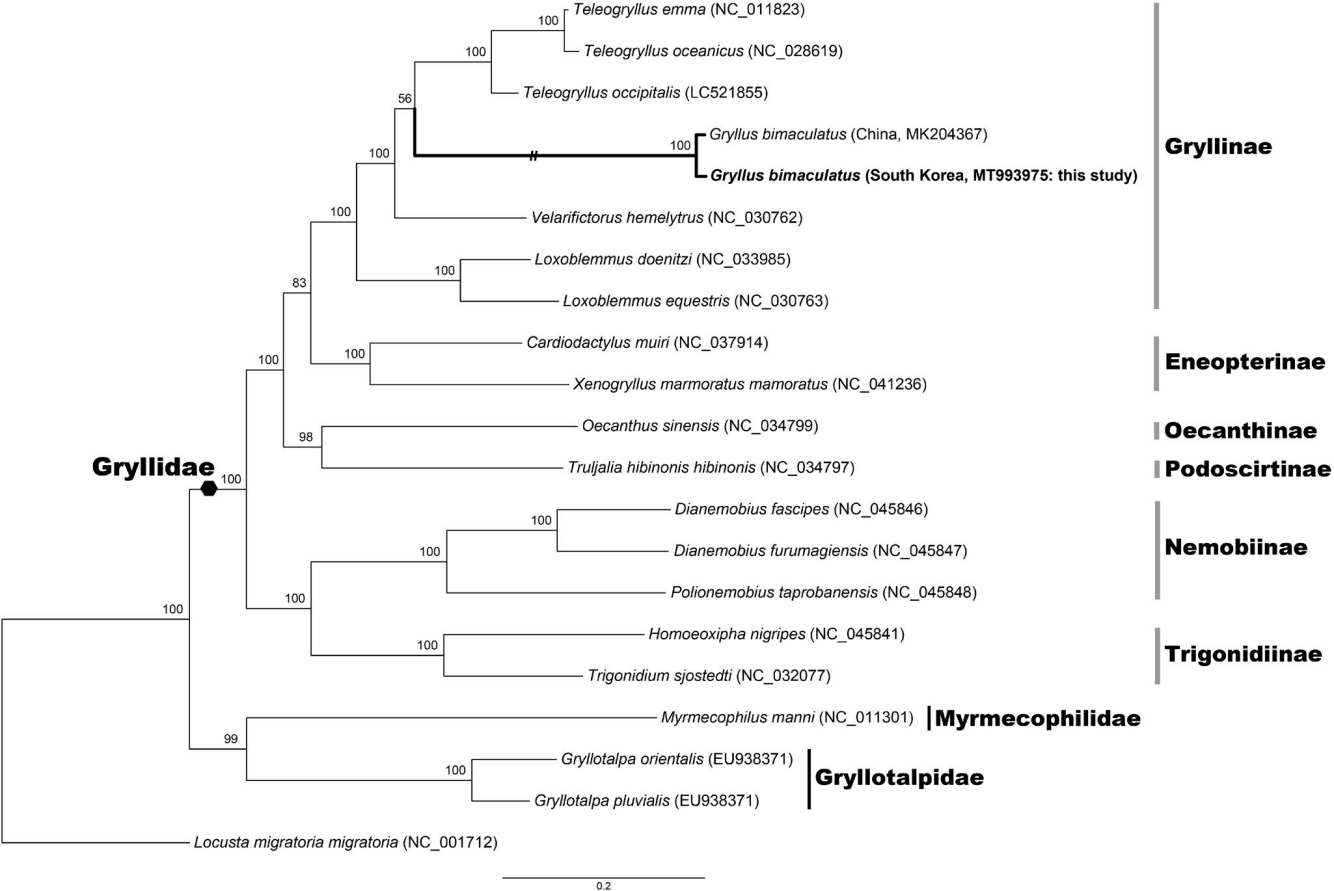
A maximum-likelihood tree reconstructed with the nucleotide sequences of 13 mitochondrial protein-coding genes (PCGs) showing relationships among 20 orthopteran species. It indicates the monophyly of the family Gryllidae, within which two specimens of *G*. *bimaculatus* from South Korea and China place together. *Locusta migratoria* (Acrididae) is used as an outgroup. Branch supports are inferred from the ultrafast bootstrap method using IQ-TREE web server (Trifinopoulos et al. 2016).

## Data Availability

The genome sequence data that support the findings of this study are openly available in GenBank of NCBI at (https://www.ncbi.nlm.nih.gov/) under the accession no. MT993975. The associated BioProject, SRA, and Bio-Sample numbers are PRJNA704187, SRR13773776, and SANB17487625, respectively.

## References

[CIT0001] Baek SY, Choi EH, Jang KH, Ryu SH, Park SM, Suk HY, Chang CY, Hwang UW. 2014. Complete mitochondrial genomes of *Carcinoscorpius rotundicauda* and *Tachypleus tridentatus* (Xiphosura, Arthropoda) and implications for chelicerate phylogenetic studies. Int J Biol Sci. 10(5):479–489. 24795529 10.7150/ijbs.8739 PMC4007361

[CIT0002] Boore JL. 1999. Animal mitochondrial genomes. Nucleic Acids Res. 27(8):1767–1780. 10101183 10.1093/nar/27.8.1767 PMC148383

[CIT0003] Harrison RG, Bogdanowicz SM. 1995. Mitochondrial DNA phylogeny of North American field crickets: perspectives on the evolution of life cycles, songs, and habitat associations. J Evolution Biol. 8(2):209–232.

[CIT0004] Kalyaanamoorthy S, Minh BQ, Wong TKF, von Haeseler A, Jermiin LS. 2017. Model finder: fast model selection for accurate phylogenetic estimates. Nat Methods. 14(6):587–589. 28481363 10.1038/nmeth.4285 PMC5453245

[CIT0005] Kim SY, Kim DI, Koo HY, Kim JE, Kim HJ, Lee YB, Kim JS, Kim HH, Han YS, Kim YC. 2020. Storage conditions and oviposition methods for *Gryllus bimaculatus* (Gryllidae) eggs. Korean J Appl Entomol. 59(2):133–138.

[CIT0006] Ma C, Li J. 2018. Comparative analysis of mitochondrial genomes of the superfamily Grylloidea (Insecta, Orthoptera) reveals phylogenetic distribution of gene rearrangements. Int J Biol Macromol. 120(Pt A):1048–1054. 30172811 10.1016/j.ijbiomac.2018.08.181

[CIT0007] Mito T, Noji S. 2008. The two-spotted cricket *Gryllus bimaculatus*: an emerging model for developmental and regeneration studies. CSH Protoc. 2008:pdb.emo110 21356736 10.1101/pdb.emo110

[CIT0008] Park SJ, Choi EH, Hwang JS, Hwang UW. 2016. The complete mitochondrial genome of a centipede *Bothropolys* sp. (Chilopoda, Lithobiomorpha, Lithobiidae). Mitochondrial DNA Part A. 27(3):2268–2269. 10.3109/19401736.2014.984174 25469812

[CIT0009] Ragge DR. 1972. An unusual case of mass migration by flight in *Gryllus bimaculatus* DeGeer (Orthoptera Gryllidae). Bull IFAN Ser A. 34:869–878.

[CIT0010] Ryu JS, Hwang UW. 2010. Complete mitochondrial genome of the longtail tadpole shrimp *Triops longicaudatus* (Crustacea, Branchiopoda, Notostraca). Mitochondrial DNA. 21(5):170–172. 20958225 10.3109/19401736.2010.503809

[CIT0011] Trifinopoulos J, Nguyen LT, von Haeseler A, Minh BQ. 2016. W-IQ-TREE: a fast online phylogenetic tool for maximum likelihood analysis. Nucleic Acids Res. 44(W1):W232–W235. 27084950 10.1093/nar/gkw256 PMC4987875

[CIT0012] Wang C, Li Q, Xu C, Liu G. 2019. The complete mitochondrial genome of two-spotted cricket *Gryllus bimaculatus* (Grylloidea: Gryllidae). Mitochondrial DNA Part B. 4(1):799–800.

[CIT0013] Wolstenholme DR. 1992. Animal mitochondrial DNA: structure and evolution. Int Rev Cytol. 141:173–216. 1452431 10.1016/s0074-7696(08)62066-5

[CIT0014] Woo HJ, Lee YS, Park SJ, Lim JT, Jang KH, Choi EH, Choi YG, Hwang UW. 2007. Complete mitochondrial genome of a troglobite millipede *Antrokoreana gracilipes* (Diplopoda, Juliformia, Julida), and juliformian phylogeny. Mol Cells. 23(2):182–191. 17464195

